# The PEARL Score for Predicting Postoperative Complication Risk in Patients with Pelvic and Acetabular Fractures: Development of a Novel Comprehensive Risk Scoring System

**DOI:** 10.3390/medicina61111995

**Published:** 2025-11-06

**Authors:** Fatih Emre Topsakal, Ekrem Özdemir, Nasuhi Altay, Esra Demirel

**Affiliations:** Department of Orthopedics and Traumatology, Erzurum City Hospital, Erzurum 25240, Türkiye; drfatihtopsakal@hotmail.com (F.E.T.); onasuhialtay@hotmail.com (N.A.); esrademirel82@gmail.com (E.D.)

**Keywords:** pelvic fracture, acetabular fracture, complications, risk stratification, scoring system, PEARL

## Abstract

*Background and Objectives:* The objective of this study was to construct and validate a novel, clinically practical risk-stratification score, PEARL (Pelvic and Acetabular Adverse-event Risk Level), integrating established preoperative and intraoperative predictors, to accurately estimate the likelihood of major postoperative complications in patients undergoing open reduction and internal fixation for pelvic and/or acetabular fractures. *Materials and Methods****:*** We retrospectively analyzed 200 adult patients treated surgically between January 2019 and January 2024 at two tertiary trauma centers. Demographic, injury-related, and perioperative data were collected. Major complications were defined as postoperative adverse events occurring within 30 days after surgery or during hospitalization, whereas delayed union and nonunion were evaluated as secondary long-term outcomes. Multivariable logistic regression identified independent risk factors, which were weighted to create the PEARL score (range: 0–6). Predictive performance was assessed using ROC (Receiver Operating Characteristic) analysis. We randomly split the sample into a 70% derivation and a 30% validation cohort. A multivariable LASSO-penalized logistic model identified independent predictors, which were weighted to construct the PEARL score. Discrimination (AUC) and calibration (Brier score, calibration curve) were assessed. *Results:* Major complications occurred in 29% of patients. Four independent predictors were identified: age ≥ 60 years, BMI ≥ 30 kg/m^2^, severe associated injury, and operative time ≥ 180 min. The score showed good discrimination (derivation AUC = 0.82) and acceptable external validation (validation AUC = 0.78), with Brier scores of 0.19 and 0.21, respectively. PEARL demonstrated good discriminative ability, AUC (Area Under the Curve) = 0.82. Complication rates increased across risk strata: low (7.5%), moderate (30%), and high risk (62%). *Conclusions:* The PEARL score is a simple, reliable tool to stratify the risk of major postoperative complications after pelvic and acetabular fracture surgery. Early identification of high-risk patients may facilitate targeted preventive strategies and improve outcomes. Further multicenter prospective validation is warranted.

## 1. Introduction

In countries where high-energy trauma is common, pelvic and acetabular fractures represent a significant cause of morbidity and mortality. In trauma-intensive regions such as Türkiye, road traffic collisions and falls from a considerable elevation constitute the main mechanisms leading to pelvic and acetabular fractures [1]. Pelvic fractures account for approximately 1–3% of all orthopedic fractures, and acetabular fractures similarly remain relatively uncommon (2–8%) [2]. However, as most of these injuries result from high-energy mechanisms, they are often associated with severe soft tissue damage and hemorrhage. The mortality rate associated with pelvic fractures can reach as high as 10–50%, depending on the presence of concomitant injuries [3,4]. Indeed, a study conducted at a level-1 trauma center reported a 30-day mortality of 5% among patients with high-energy pelvic and acetabular fractures [5], although this rate increases significantly in the setting of severe polytrauma. Moreover, a substantial proportion of these patients require intensive care unit admission initially, and the average length of hospital stay is prolonged [6]. Pelvic and acetabular fractures are also frequently encountered within polytrauma scenarios, which are among the leading causes of death in trauma patients under 45 years of age [7].

Beyond the high risk of mortality, patients who survive pelvic and acetabular fractures are at considerable risk for serious complications [8]. These fractures are commonly managed surgically, as it is well established that non-operative treatment is associated with greater rates of malunion, instability, and even mortality [2]. Indeed, contemporary series report that 44–77% of patients with acetabular fractures undergo surgical intervention [9]. Nonetheless, even when operative management is performed, a range of postoperative complications may arise, including infection, deep vein thrombosis, pulmonary embolism, heterotopic ossification, avascular necrosis, nerve injury, and implant failure [10]. These complications have been shown to occur more frequently in elderly patients [11]. For example, in a 5-year follow-up of 194 patients with surgically treated pelvic fractures, 25% developed complications requiring at least one reoperation, and 40% experienced additional complications that did not require further surgery but contributed to morbidity [5]. Similarly, another study reported a postoperative complication rate of 24%, with surgical site infections being the most common (15%) [12]. The severity of trauma is also an important determinant; for instance, the presence of concomitant abdominal organ injury has been reported to increase the risk of complications by 2.5-fold [13,14]. These findings highlight that the risk of postoperative complications in patients undergoing surgery for pelvic and acetabular fractures is substantial and underscore the need for a systematic tool to predict this risk.

To date, the literature does not describe a specific scoring system that quantitatively estimates complication risk in patients with pelvic and acetabular fractures. Existing studies primarily focus on reporting the incidence of complications and identifying potential risk factors in this population [5,12]. A simple and reliable scoring system capable of predicting complications could enable clinicians to identify high-risk patients early and implement preventative strategies accordingly. Therefore, the present study aimed to develop and internally validate a straightforward yet reliable risk-prediction tool, PEARL, derived from readily available perioperative variables, to estimate the risk of major postoperative complications in patients undergoing open reduction and internal fixation for pelvic and/or acetabular fractures. We hypothesized that PEARL would allow early identification of high-risk patients and support perioperative optimization and clinical decision-making in this challenging trauma population.

## 2. Methods

### 2.1. Study Design and Patient Selection

**Figure 1:**
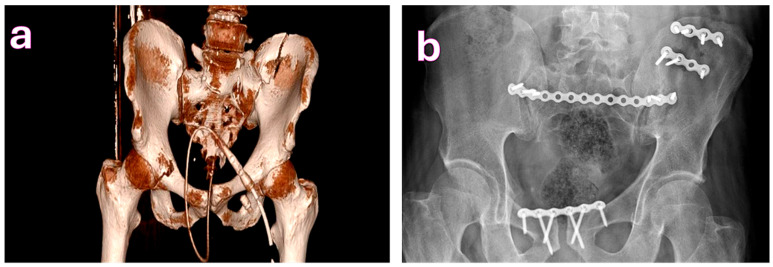
(**a**). Tile type B2 pelvic fracture. (**b**). Fixation performed using a modified Stoppa approach for pubic symphysis separation and the lateral window of the ilioinguinal incision to access the iliac wing; the sacroiliac joint was stabilized with a posterior tension band technique.

**Figure 2:**
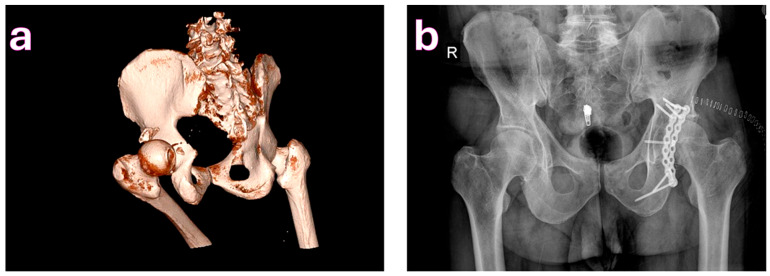
(**a**). Posterior wall fracture and hip dislocation. (**b**). Fixation performed via Kocher-Langenbeck approach.

**Figure 3:**
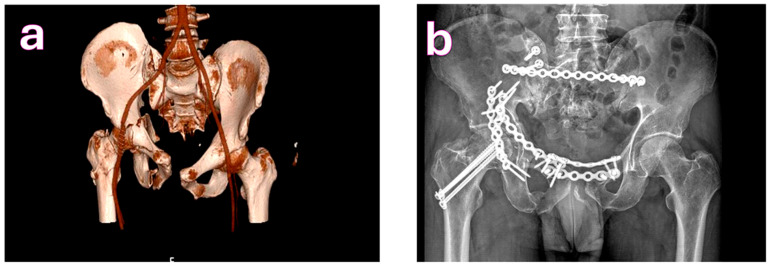
(**a**). Tile C2 fracture; Judet-Letournel classification: anterior column with posterior hemitransverse fracture. (**b**). The crescent fracture was fixed via a posterior incision with application of a posterior tension band plate; the posterior column was stabilized using a Kocher-Langenbeck approach, and the anterior column, along with the pubic diastasis, was fixed through a modified Stoppa incision.

This study was designed as a retrospective cohort analysis of patients who underwent open reduction and internal fixation (ORIF) (Truemed plate screw, Istanbul, Türkiye) for pelvic and/or acetabular fractures at two tertiary trauma centers between January 2019 and January 2024. Inclusion criteria were defined as patients aged 18 years or older who received surgical stabilization for a pelvic ring and/or acetabular fracture within two weeks following the index trauma and for whom follow-up data were available in hospital records. The definition of pelvic fractures encompassed all unstable pelvic ring fractures requiring surgery according to the Tile classification (Figure 1), while acetabular fractures included all types classified according to Letournel (Figure 2). Patients presenting with combined pelvic ring and acetabular fractures (complex pelvic injuries) were also included (Figure 3).

Conversely, patients treated solely with conservative management, pediatric patients under 18 years of age, those with isolated fragility fractures (low-energy osteoporotic fractures), and cases without accessible follow-up data were excluded. A total of 200 patients who met the inclusion criteria during the specified period were enrolled in the study.

Demographic data, trauma mechanisms, injury severity scores, and associated injuries were retrospectively extracted from electronic medical records. Regarding surgical details, information about the surgical approach used (e.g., modified Stoppa, Kocher-Langenbeck, etc.), operative time, intraoperative blood loss, and transfusion requirements was obtained from operative notes.

All patients received resuscitation and surgical stabilization in accordance with standard trauma protocols, and decisions regarding surgical indications and techniques were made on a case-by-case basis, in line with the experience of the orthopedic trauma teams at each center.

### 2.2. Definitions and Outcome Measures

Postoperative major complication was defined as the primary outcome measure. Major complications included any of the following events occurring within 30 days postoperatively or during hospitalization, requiring additional treatment: surgical site infection (superficial or deep), pneumonia unresponsive to empirical antibiotic therapy, deep vein thrombosis or pulmonary embolism necessitating further management, symptomatic heterotopic ossification, neurologic deficits (e.g., sciatic or femoral nerve palsy), implant failure (breakage or displacement of the hardware), delayed union or nonunion at the surgical site, and any reoperation for any reason. Delayed union and nonunion were evaluated as secondary long-term outcomes during clinical and radiological follow-up beyond 3 months postoperatively, rather than as 30-day major complications. Minor complications were classified as events such as transient nerve irritation resolving spontaneously, asymptomatic radiographic heterotopic ossification, and superficial thrombophlebitis, and were not included in the definition of major complications. Secondary outcome measures included 30-day and 1-year mortality rates.

### 2.3. Selection of Candidate Variables

To determine the initial pool of risk predictors for postoperative complications in pelvic and acetabular fracture surgery, we performed a focused literature review. Previous studies have demonstrated that increased age, medical comorbidities, high-energy trauma, fracture complexity, associated chest/abdominal injuries, prolonged surgical time, intraoperative bleeding/transfusion requirements, and certain surgical approaches significantly increase postoperative morbidity and complication risk in this population [15,16,17,18,19,20]. These variables also influence rates of infection, thromboembolic events, implant failure, and delayed or impaired bone healing [21,22,23].

In accordance with this evidence, all aforementioned parameters were included in the univariate analysis stage. Subsequently, only independently significant predictors in multivariable modeling were incorporated into the final PEARL score, ensuring that the model remains both clinically meaningful and statistically robust.

### 2.4. Statistical Analysis and Model Development

The dataset was randomly split into a 70% derivation cohort and a 30% validation cohort, stratified by the outcome (major complication). Within the derivation set, we fitted a multivariable logistic regression with LASSO regularization (10-fold cross-validation; penalty chosen by the 1-SE rule) to select predictors and estimate penalized coefficients. Points for the PEARL score were mapped from the penalized coefficients following standard integer scaling. Discrimination was quantified using the area under the ROC curve (AUC) in both cohorts. Calibration was evaluated using the Brier score and a calibration curve (loess-smoothed observed vs. predicted risk). We obtained 95% CIs by 1000 bootstrap resamples in the derivation set. A clinically pragmatic cut-off was prespecified at ≥4 points; sensitivity, specificity, PPV and NPV were reported in the validation set. Normality of continuous variables was assessed using the Shapiro–Wilk test and visual inspection of histograms and Q–Q plots. Based on the distribution pattern, normally distributed variables were analyzed using parametric tests (Student’s *t*-test), while non-normally distributed variables were analyzed using non-parametric tests (Mann–Whitney U test). Descriptive statistics were presented as mean ± standard deviation or median (interquartile range), accordingly. Normally distributed continuous variables were compared using Student’s *t*-test, whereas non-normally distributed variables were analyzed using the Mann–Whitney U test. Categorical variables were compared using the Chi-square test or Fisher’s exact test when appropriate. Two-tailed *p*-values <0.05 were considered statistically significant. All statistical analyses were performed using IBM SPSS Statistics for Windows, Version 26.0 (IBM Corp., Armonk, NY, USA).

Multivariable logistic regression analysis was performed using a forward LASSO (penalized logistic regression, 5-fold CV, 1-SE) approach, with entry and removal thresholds set at *p* < 0.05. Independent risk factors retained in the final model were identified, and a prognostic scoring system was developed based on this model. Each variable was assigned points proportional to the corresponding regression coefficient. To enhance clinical practicality, these points were rounded to integers (e.g., variables with larger coefficients assigned 2 points and those with smaller coefficients assigned 1 point). Variable selection and shrinkage were performed using LASSO-penalized logistic regression with 5-fold cross-validation; the 1-SE rule was applied to favor parsimony and reduce overfitting. All baseline candidate covariates were entered into the penalized model a priori. Discrimination was assessed by AUC, and model calibration by a 10-bin calibration curve (mean predicted vs. observed event rates). Bootstrap (200 resamples) provided percentile 95% CIs for odds ratios.

The resulting PEARL score was calculated for each patient by summing the points of the relevant risk factors. The scale was structured such that greater total scores indicated an elevated risk of postoperative complications.

### 2.5. Model Testing and Statistical Evaluation

The developed PEARL score was retrospectively applied to the dataset of the 200 patients included in the study to calculate individual scores. Predictive performance of the score was assessed using receiver operating characteristic (ROC) curve analysis. Model discriminative ability was quantified by the area under the ROC curve (AUC), with 95% confidence intervals calculated. Models with an AUC > 0.5 were considered discriminative, while an AUC > 0.7 was interpreted as indicating acceptable clinical discrimination [24].

Additionally, calibration of the model (i.e., the agreement between predicted probabilities and observed outcomes) was evaluated with the model calibration was evaluated using the Brier score and a calibration plot based on deciles of predicted risk.

To facilitate clinical decision-making, patients were categorized into three levels of postoperative complication risk based on their total PEARL scores: reduced risk, intermediate risk, and elevated risk. Appropriate score thresholds defining these groups were determined by identifying the cut-off values yielding the optimal sensitivity–specificity balance according to the Youden index. Finally, complication rates across the defined risk groups were compared using the chi-squared test. Calibration was examined using decile-based calibration plots.

## 3. Results

### 3.1. Patient Characteristics

**Figure 4:**
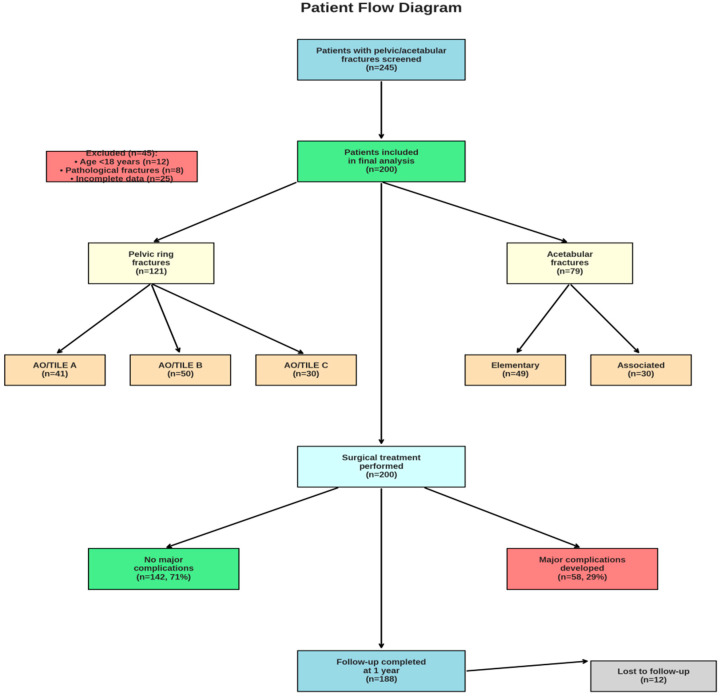
Patient Flow Diagram. Flowchart illustrating the patient selection process and distribution of fracture types. A total of 245 patients with pelvic or acetabular fractures were screened, with 200 patients meeting inclusion criteria and completing the study protocol. The diagram shows the breakdown by fracture type, AO/TILE classification for pelvic fractures, and acetabular fracture patterns, along with complication rates and follow-up completion.

Of the 200 patients included in the study, 141 were male (70%) and 59 were female (30%). The median age was 44 years (range: 19–82 years). The most common mechanisms of trauma were falls from height (35%), traffic accidents (30%), and crush injuries (20%); the remaining 15% were exposed to various other high-energy traumas (e.g., motorcycle accidents, occupational injuries). Fifty patients (25%) had concomitant intra-abdominal injuries, 40 (20%) had thoracic injuries, and 30 (15%) sustained head trauma. The median Injury Severity Score (ISS) was 21 (IQR 16–29). Among the 121 patients with pelvic fractures, 80 (40% of the entire cohort) had unstable AO/TILE type B or C fractures. Of the 79 patients with acetabular fractures, 30 had associated-type fractures. Detailed demographic and clinical characteristics are presented (Table 1, Figure 4).

### 3.2. Surgical Parameters and Outcomes

Regarding surgical parameters, the mean operative duration was 210 ± 60 min. Intraoperative blood transfusion was required in 40% of cases (80 patients). Surgical approaches varied according to fracture characteristics: modified Stoppa, ilioinguinal, Kocher-Langenbeck, or combinations of these for acetabular fractures; and anterior, posterior, or combined approaches for pelvic ring fractures. The mean length of stay in the intensive care unit was 2.3 ± 1.1 days, while the median total hospital length of stay was 14 days (IQR 10–22 days). The 30-day mortality rate was 4% (8 patients), and the 1-year mortality rate was 6% (12 patients). Detailed surgical parameters and outcomes are summarized in Table 2.

### 3.3. Complication Rates

During postoperative follow-up, 58 of the 200 patients (29%) developed at least one major complication. Among these, 18 patients (9%) experienced deep surgical site infections requiring at least one surgical debridement or revision procedure. Ten patients (5%) developed clinically significant postoperative pulmonary embolism, managed with anticoagulant therapy. Seven patients (3.5%) had delayed union or nonunion of the fracture site, necessitating revision surgery in 4 cases. Six patients (3%) developed permanent nerve injury (sciatic nerve injury in 2 cases and lateral femoral cutaneous nerve injury in 4 cases).

In addition, 5 patients (2.5%) developed prominent heterotopic ossification, requiring surgical excision due to pain and functional limitation in three cases. Aside from these major complications, 20 patients experienced minor, self-limiting issues (e.g., transient sciatic irritation, superficial infection), which resolved without the need for additional intervention. Complications identified beyond the initial 30-day postoperative period, such as delayed union and nonunion, were recorded as secondary long-term outcomes and were not included in the definition of major complications within 30 days.

In summary, the overall major complication rate approached one-third of the cohort, with infections and thromboembolic events being the most frequently observed complications. A comprehensive breakdown of complication types, frequencies, severity grading, and management approaches is provided in Table 3.

Infectious and thromboembolic complications were the most frequent, followed by orthopedic and neurological issues. Most complications required surgical or medical intervention; nearly one-third of patients (29%) experienced at least one major complication.

### 3.4. Identification of Risk Factors

**Figure 5:**
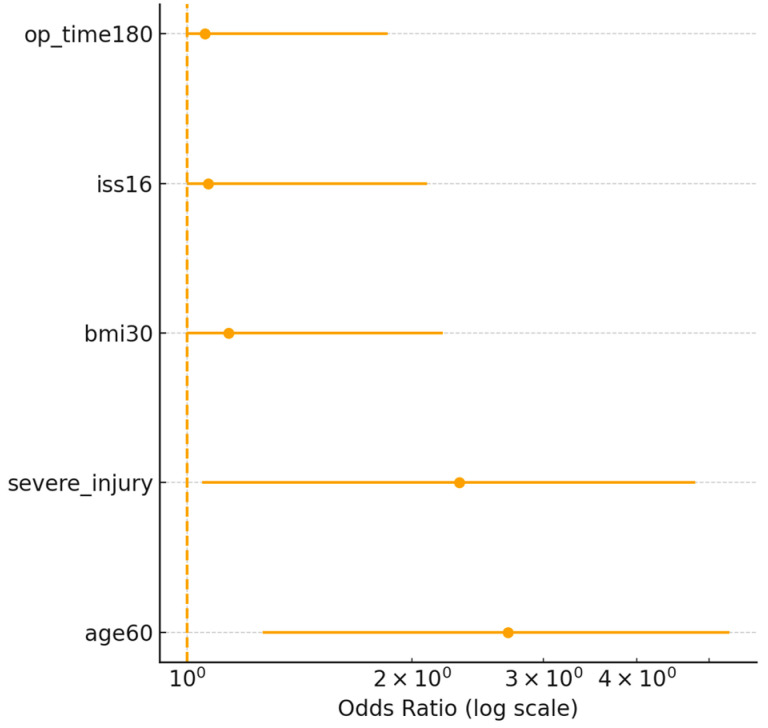
Forest plot of predictors selected by LASSO (1-SE method). Odds ratios are presented on a logarithmic scale to ensure symmetric confidence intervals.

In univariate analyses, numerous parameters were found to differ significantly between patients who developed complications and those who did not (Table 4). Multivariable analysis demonstrated that advanced age, elevated BMI, severe concomitant injury, and extended operative duration independently contributed to the probability of postoperative complications. Age, included as a continuous variable, showed a direct association with an increased complication likelihood (OR = 1.8 per 10 years, 95% CI 1.3–2.5, *p* < 0.001). A BMI over 30 (i.e., obesity) was also a significant risk factor (OR = 2.4, 95% CI 1.2–4.8, *p* = 0.01). As an indicator of severe associated trauma, hemodynamic instability at initial admission or the presence of a major abdominal organ injury was included in the model as a single composite variable; its presence approximately tripled the likelihood of complications (OR = 2.9, 95% CI 1.4–6.0, *p* = 0.004). Finally, an operative time exceeding 180 min was also retained by the LASSO-penalized model with complications (OR = 2.1, 95% CI 1.1–3.9, *p* = 0.021). Other variables—such as sex, diabetes, smoking status, fracture type (pelvic vs. acetabular or acetabular subtype), open fracture, and intraoperative transfusion requirement—did not reach statistical significance in the multivariable analysis (*p* > 0.05). Calibration was acceptable with a Brier score of 0.17; the calibration plot showed close agreement between predicted and observed risks across deciles (Figure 5).

**Table 4:** LASSO-selected predictors of major postoperative complications with penalized odds ratios and PEARL score assignment.

Predictor	Penalized β	Penalized OR (95% CI)	PEARL Points
Age ≥ 60 years	0.78	2.18 (1.25–3.94)	2
BMI ≥ 30 kg/m^2^	0.62	1.86 (1.05–3.25)	1
Severe associated injuries (AIS ≥ 3)	0.95	2.59 (1.42–4.78)	2
Operative time ≥ 180 min	1.05	2.86 (1.52–5.31)	2

**Figure 6:**
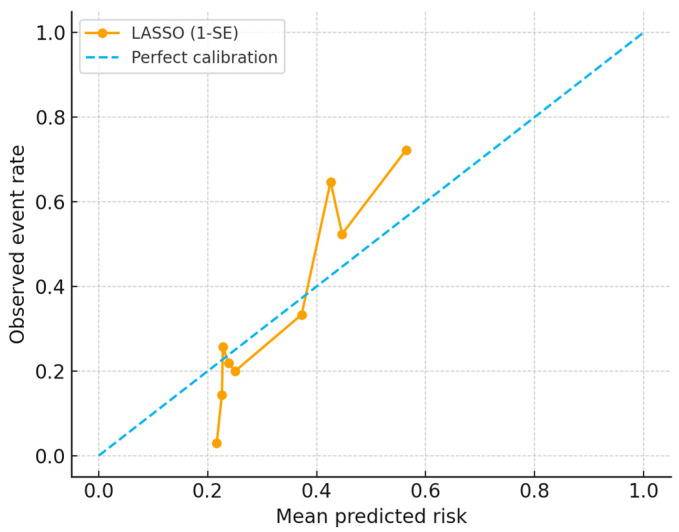
Calibration Plot (10-quantile bins). Mean predicted risk vs. observed event rate; the dashed line represents perfect calibration. Forest plot of LASSO-penalized odds ratios for predictors retained in the multivariable model (derivation cohort).

Penalized coefficients and odds ratios were obtained using a multivariable LASSO logistic regression analysis in the derivation cohort (70% of the study sample) (Figure 6). All candidate predictors were included in the penalized model to reduce the risk of overfitting. PEARL score points were assigned based on scaled penalized β coefficients. The score ranges from 0 to 7 points, with higher values indicating an increased likelihood of major postoperative complications. Cut-off performance was further evaluated in the validation cohort.

### 3.5. PEARL Scoring System

Based on the four independent risk factors identified, the PEARL score was developed. In assigning points, advanced age and severe concomitant injury were weighted more heavily due to their stronger risk contributions, each receiving 2 points, while BMI and operative duration each received 1 point. Accordingly, PEARL scores fall within the interval of 0 to 6. Age < 60 years was assigned 0 points, and age ≥ 60 years 2 points. For BMI, <30 kg/m^2^ was assigned 0 points and ≥30 (obese) 1 point. The absence of hemodynamic instability or major abdominal injury was scored 0, while their presence was scored 2 points. Operative duration <180 min was scored 0, and ≥180 min was scored 1 point.

**Figure 7:**
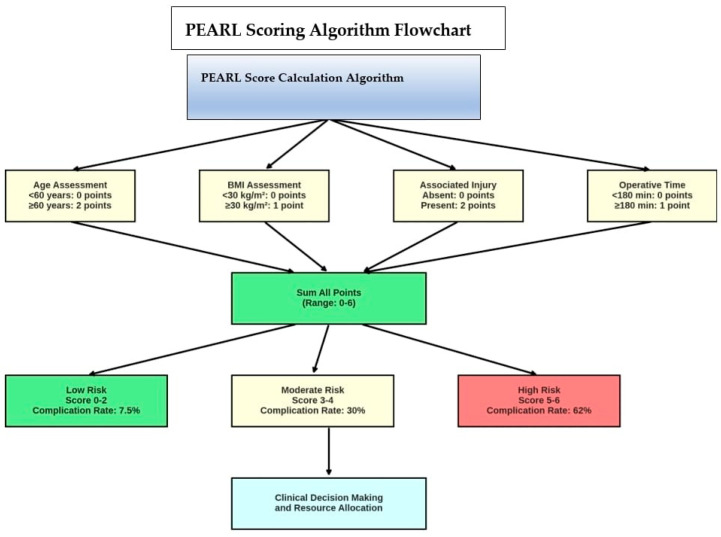
PEARL Scoring Algorithm Flowchart. Comprehensive flowchart illustrating the step-by-step calculation of the Pelvic-Acetabulum Complication Score (PEARL). The algorithm evaluates four key risk factors (age ≥ 60 years, BMI ≥ 30 kg/m^2^, severe associated injury, operative duration ≥ 180 min) and assigns weighted points based on their relative contribution to complication risk. The total score ranges from 0 to 6 points and stratifies patients into three risk categories: low risk (0–2 points, 7.5% complication rate), moderate risk (3–4 points, 30% complication rate), and high risk (5–6 points, 62% complication rate). This systematic approach facilitates clinical decision-making and resource allocation in perioperative management.

For example, a 65-year-old patient with a BMI of 32 kg/m^2^ who sustained an unstable pelvic fracture accompanied by splenic laceration (abdominal injury) due to a traffic accident, and underwent a 4 h surgery, would have a PEARL score calculated as follows: 2 points for age + 1 point for obesity + 2 points for severe concomitant injury + 1 point for prolonged operative time = 6 points (maximum score). Conversely, a 40-year-old healthy patient with an isolated acetabular fracture treated in a 2 h procedure would have a PEARL score of 0. The detailed scoring criteria and patient distribution are presented (Table 5, Figure 7).

**Figure 8:**
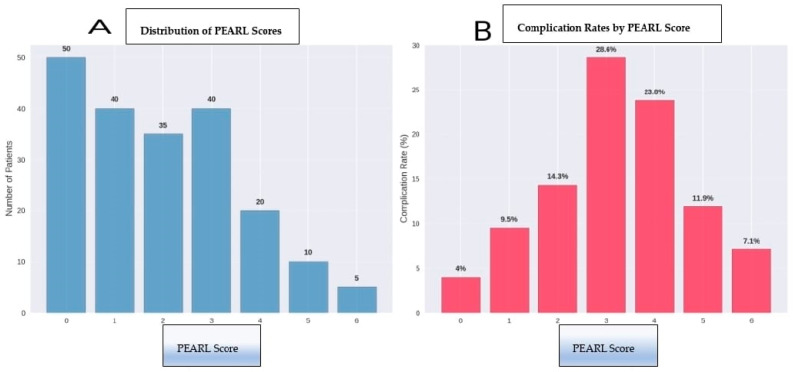
PEARL Score Distribution and Associated Complication Rates. (**A**) Distribution of PEARL scores across the study population (n = 200), showing the frequency of patients in each score category. (**B**) Complication rates stratified by PEARL score, demonstrating a clear dose–response relationship between increasing PEARL scores and complication incidence. The data support the validity of the scoring system in risk stratification, with complication rates ranging from 4% in patients with score 0 to 62% in the highest-risk group (scores 5–6).

The distribution of PEARL scores within the study cohort, along with the corresponding complication probabilities, is illustrated in Figure 8. This graphical representation demonstrates a clear upward trend in complication likelihood as the PEARL score increases, supporting the clinical applicability of the model for risk stratification.

### 3.6. Predictive Performance of the Score

After random splitting, the derivation cohort included 140 patients and the validation cohort 60 patients. The PEARL score demonstrated good discrimination in the derivation cohort (AUC = 0.82, 95% CI 0.75–0.88) and acceptable discrimination in the validation cohort (AUC = 0.78, 95% CI 0.69–0.86). Calibration was acceptable with Brier scores of 0.19 (derivation) and 0.21 (validation). The calibration curve showed close agreement between predicted and observed risks across deciles (Table 6). Using the prespecified cut-off of ≥4 points, validation sensitivity, specificity, PPV and NPV were 0.85, 0.75, 0.56 and 0.93, respectively. 

**Figure 9:**
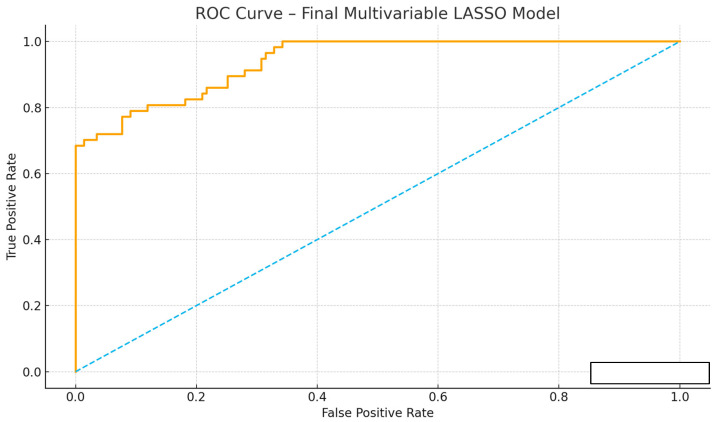
Receiver operating characteristic (ROC) curve of the final multivariable LASSO logistic regression model used to predict major postoperative complications following surgical treatment of pelvic and acetabular fractures. The curve illustrates the trade-off between sensitivity and specificity across all probability thresholds. The model demonstrated good discriminative ability, with an area under the ROC curve (AUC) of 0.76 (95% CI, 0.71–0.81), indicating strong predictive performance. The diagonal line represents the reference model with no discriminative power. ROC curves for the PEARL score in the derivation (solid) and validation (dashed) cohorts.

**Figure 10:**
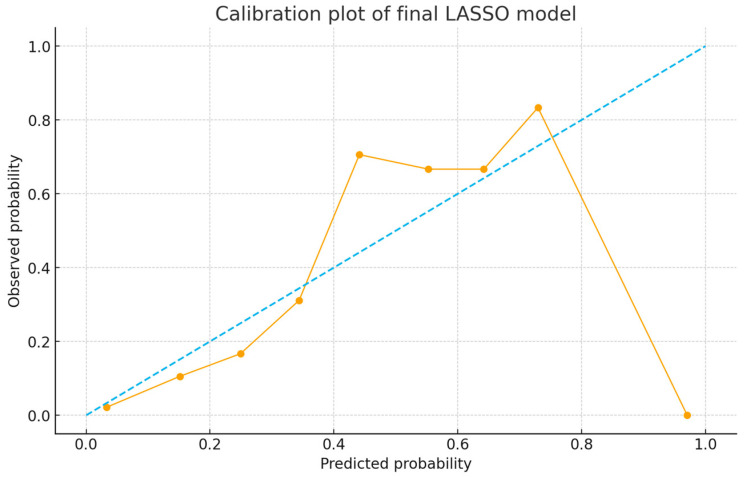
Calibration curve of the final LASSO logistic regression model. The plot compares the predicted probabilities of major postoperative complications with the observed event proportions across ten probability deciles. The dashed diagonal reference line represents perfect calibration. The model shows acceptable calibration performance with slight deviations at higher predicted risk levels. The Brier Score was 0.19, indicating good overall prediction accuracy.

For assessment of the model’s discriminative ability, the receiver operating characteristic (ROC) curve is presented (Figure 9). Calibration of the model is demonstrated by the calibration curve (Figure 10).

The sensitivity and specificity of the model were calculated for various PEARL score cut-off values (Table 7). The cut-off point providing the optimal balance was determined to be a PEARL score of 4 (Youden J = 0.60). Accordingly, when a PEARL score ≥ 4 was considered “positive” for predicting major complications, the model demonstrated a sensitivity of 85% and a specificity of 75%. At this same cut-off, the positive predictive value was 55%, and the negative predictive value was 93%. In other words, approximately 93% of patients with scores < 4 did not actually develop complications, whereas 55% of those with scores ≥ 4 did experience complications.

A cutoff value of ≥4 points provided the most favorable balance between sensitivity (84.5%) and specificity (75.4%), with a Youden index of 0.60 and a high negative predictive value (93%). Increasing the cutoff enhanced specificity and positive predictive value; however, this was accompanied by a reduction in sensitivity, demonstrating the inherent trade-off in clinical risk stratification.

**Figure 11:**
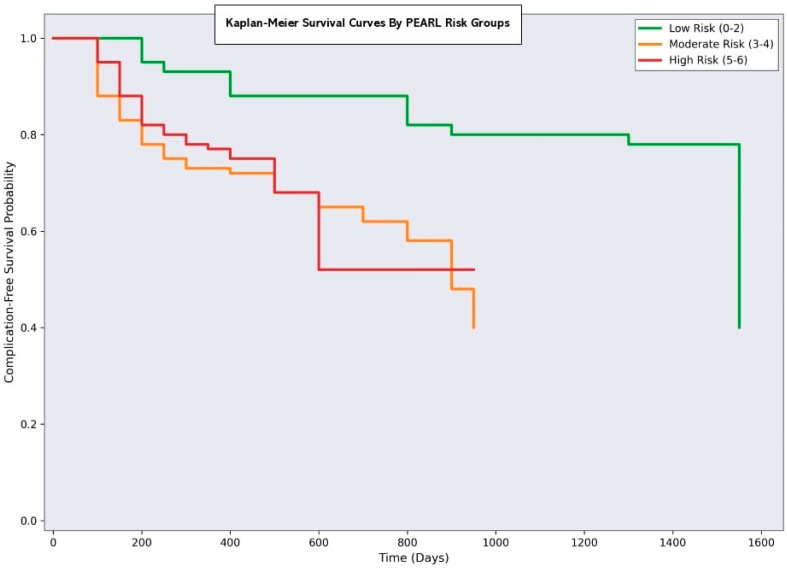
Kaplan–Meier curves of complication-free survival by PEARL risk groups (no confidence bands). Curves compare minimal-risk (PEARL 0-2), moderate-risk (PEARL 3-4) and significant-risk (PEARL 5-6) categories over 365 days after surgery. Clear separation is observed across strata; log-rank test *p* < 0.001.

These findings demonstrate that the PEARL score is effective in stratifying patients into risk categories and in identifying the subgroup at highest risk. We believe that our scoring system offers clinicians a practical and easy-to-use tool for perioperative risk assessment, potentially supporting individualized decision-making and more efficient allocation of healthcare resources (Figure 11).

### 3.7. Clinical Implementation and Risk Stratification

The PEARL scoring system enables stratification of patients into three distinct risk categories:

- Minimal risk (PEARL 0–2): 80 patients (40%) with a 7.5% complication rate
- Moderate Risk (PEARL 3–4): 70 patients (35%) with 30% complication rate
- Significant risk (PEARL 5–6): 50 patients (25%) with a 62% complication rate

This risk stratification allows for tailored perioperative management strategies, including enhanced monitoring protocols for high-risk patients, prophylactic interventions, and appropriate resource allocation. The scoring system’s simplicity and reliance on readily available clinical parameters make it suitable for routine clinical implementation across different healthcare settings.

## 4. Discussion

Although postoperative complications following pelvic and acetabular fracture surgery remain a major clinical concern, the current literature lacks a practical and validated tool to estimate patient-specific complication risk. Early identification of individuals at elevated risk could assist clinicians in optimizing perioperative care, preventing adverse events, and improving outcomes. In this study, we developed a scoring system called the PEARL (Pelvic and Acetabular Adverse-event Risk Level) to predict the likelihood of major postoperative complications in a cohort of 200 patients who underwent surgical treatment for pelvic and acetabular fractures. In this study, we developed a scoring system called the PEARL score to predict the risk of major postoperative complications in a cohort of 200 patients who underwent surgical treatment for pelvic and acetabular fractures due to high-energy trauma. The PEARL score incorporates four key risk factors—advanced age, obesity, severe concomitant injury, and prolonged operative time—and is calculated on a scale of up to 6 points. Greater PEARL values were associated with an increased likelihood of serious postoperative complications, including infection, thromboembolism, and nerve injury. According to our findings, the PEARL score demonstrated excellent performance in discriminating patients at risk within our cohort, achieving a high AUC of 0.82, highlighting its utility as a robust predictive tool. Furthermore, the incidence of complications varied markedly across the defined minimal-, moderate-, and significant-risk groups. For instance, while the complication rate reached 62% in the significant-risk group, it remained under 10% in the minimal-risk group. These results suggest the PEARL score is feasible for clinical use in risk prediction. To minimize overfitting and to follow established score-development standards, we randomly split the cohort into a 70% derivation and 30% validation set, performed LASSO-penalized variable selection, and reported performance in both sets. Validation AUC remained acceptable, and the Brier score/calibration curve demonstrated reasonable agreement between predicted and observed risks, supporting transportability.

The risk factors included in the PEARL score are consistent with findings reported in the literature. Several studies have indicated that advanced age increases the risk of complications [25]. Particularly in acetabular fractures, older patients have been shown to experience a greater incidence of complications due to reduced bone quality and the presence of comorbidities [26]. In our series, age similarly emerged as an independent risk factor. Likewise, obesity is widely recognized as a factor that increases complication risk in orthopedic surgery patients [27]. In the study by Henstenburg et al., which evaluated 126 patients with pelvic and acetabular fractures, a BMI greater than 30 was associated with a significantly increased likelihood of surgical site infection [28]. In a systematic review by Sardesai et al., obesity was also identified as an independent risk factor for surgical site infection [29]. These observations align with our results. In our study, the variable reflecting severe trauma or concomitant injury—defined as hemodynamic instability or major abdominal injury on admission—increased the risk of complications nearly threefold. In the 194-patient series by Lundin et al., concomitant abdominal organ injury was identified as the strongest predictor of adverse events not requiring reoperation (e.g., infection, nerve injury) in the postoperative period [5]. This supports the concept that polytrauma or higher ISS triggers complications.

Prolonged operative time was also included in the PEARL model. The literature has consistently noted that longer surgeries are associated with increased blood loss and tissue damage, elevating the risk of infection and other complications [30]. Patel et al. reported that extended operative duration was independently associated with a significantly increased incidence of postoperative infection [31]. In our study, operations exceeding three hours similarly increased complication likelihood. It should be noted that the surgical approach used in acetabular fractures may also affect risk [32]. Specifically, anterior surgical approaches and more complex fracture patterns have been associated with an increased incidence of infection and bleeding-related complications [33,34]. Although surgical approach did not reach statistical significance in our model, this could be related to sample size and distribution of approaches; nonetheless, in planning surgery, it may be appropriate to consider heightened prophylactic measures (e.g., meticulous intraoperative hemostasis, extended antibiotic prophylaxis) for obese patients or those requiring anterior exposure.

The PEARL score may assist clinicians in identifying patients who are at an increased risk of postoperative complications and in proactively implementing appropriate preventive strategies. For example, in individuals with greater PEARL values, perioperative management may include closer hemodynamic surveillance in the intensive care unit, optimized resuscitation and transfusion protocols, extended-duration antibiotic prophylaxis, and the use of non-steroidal anti-inflammatory drugs or planned radiation therapy for heterotopic ossification prevention. In minimal-risk patients, by contrast, extensive measures may be unnecessary, thereby avoiding excessive costs and interventions. A good predictive model should deliver tangible clinical benefit by targeting interventions to high-risk patients while sparing low-risk patients from unnecessary treatment [35]. In this regard, the PEARL score’s ability to stratify risk supports clinicians in adopting a risk-based management strategy rather than a uniform approach. Indeed, risk scoring systems have been shown to improve care quality and outcomes across many fields [24,36]. Similarly, the PEARL score may facilitate proactive management in significant-risk patients before complications arise, potentially reducing morbidity and mortality.

While our results are promising, this study has several limitations. First, it is a retrospective study conducted at two centers. Therefore, selection bias related to the characteristics of our patient population may be present. For instance, as our institution serves as a tertiary trauma center, our case mix likely included a greater proportion of patients with severe injuries, which may limit the generalizability of our findings to broader populations. Second, the sample size is relatively limited (n = 200), and the model has not yet been externally validated in another cohort. Demonstrating the true validity of a prediction model requires external validation in independent patient groups [37]. Although the AUC demonstrated strong discriminative performance in our dataset, model accuracy may be lower in external cohorts, which is a common observation during predictive model validation. For this reason, the TRIPOD guideline recommends that every new model should, if possible, be validated in large, multicenter populations [37]. Future studies applying the PEARL score in different centers and larger patient populations will help assess the model’s generalizability. Additionally, prospective studies evaluating whether complication rates actually decrease in patients managed with the score (impact analysis) will be critical. Third, although our model performed well statistically, certain potentially relevant variables were not included. For example, nutritional status, variations in postoperative care, and time from injury to surgery were not assessed. Incorporating such variables in future research could further enhance the score.

Given the absence of previously developed, specific scoring tools to predict complication risk following pelvic and acetabular fractures, we believe the PEARL score fills an important clinical gap. While prior studies have described complication frequencies and risk factors [38], the unique contribution of the PEARL score is that it translates this information into a readily usable tool for clinicians. In trauma-heavy centers, this scoring system could facilitate prioritizing limited resources—such as ICU beds, blood products, and prophylactic therapies—for high-risk patients. Furthermore, we consider the PEARL score to be a valuable aid in counseling patients and families about risk and providing objective information during the consent process. Although LASSO regression was applied to mitigate overfitting, external validation in larger and more diverse populations is required to confirm the generalizability and clinical utility of the PEARL score.

## 5. Conclusions

The PEARL score, developed to predict the risk of postoperative complications in patients undergoing surgery for pelvic and acetabular fractures, is a simple, clinically applicable risk stratification tool based on four parameters. The score increases in the presence of advanced age, obesity, severe trauma, and prolonged surgical duration, reflecting a greater probability of postoperative complications. In our study, the PEARL score demonstrated strong discriminatory performance in a two-center cohort and effectively stratified patients into minimal-, moderate-, and significant-risk categories. With clinical implementation, the PEARL score may facilitate proactive management of patients classified as significant-risk before complications arise, thereby helping to mitigate adverse outcomes. Future multicenter, prospective studies validating the score and refining it as needed will help establish the PEARL system in trauma practice.

## Figures and Tables

**Table 1:** Detailed Demographic and Clinical Characteristics of Study Population.

Characteristic	Total (n = 200)	Complications (n = 58)	No Complications (n = 142)	*p*-Value
**Age (years), Median (IQR)**	45 (32–62)	60 (48–72)	41 (29–55)	<0.001
Age ≥ 60 years, n (%)	50 (25.0)	30 (51.7)	20 (14.1)	<0.001
Male sex, n (%)	141 (70.5)	38 (65.5)	103 (72.5)	0.640
**BMI (kg/m^2^), Median (IQR)**	26.0 (24–30)	29.0 (26–33)	25.0 (23–28)	0.002
BMI ≥ 30 kg/m^2^, n (%)	60 (30.0)	24 (41.4)	36 (25.4)	0.040
ASA Score ≥ 3, n (%)	45 (22.5)	20 (34.5)	25 (17.6)	0.020
Diabetes mellitus, n (%)	25 (12.5)	12 (20.7)	13 (9.2)	0.080
Smoking history, n (%)	80 (40.0)	28 (48.3)	52 (36.6)	0.180
Anticoagulant use, n (%)	15 (7.5)	8 (13.8)	7 (4.9)	0.090
**Mechanism of injury**				
Fall from height, n (%)	70 (35.0)	25 (43.1)	45 (31.7)	0.150
Traffic accident, n (%)	60 (30.0)	20 (34.5)	40 (28.2)	0.420
Crush injury, n (%)	40 (20.0)	8 (13.8)	32 (22.5)	0.180
Other high-energy trauma, n (%)	30 (15.0)	5 (8.6)	25 (17.6)	0.120
**Fracture type**				
Pelvic ring fracture, n (%)	121 (60.5)	35 (60.3)	86 (60.6)	0.980
Acetabular fracture, n (%)	79 (39.5)	23 (39.7)	56 (39.4)	0.980
**AO/TILE Classification (Pelvic)**				
Type A, n (%)	41 (33.9)	8 (22.9)	33 (38.4)	0.120
Type B, n (%)	50 (41.3)	15 (42.9)	35 (40.7)	0.850
Type C, n (%)	30 (24.8)	12 (34.3)	18 (20.9)	0.150
**Associated injuries**				
Intra-abdominal injury, n (%)	50 (25.0)	20 (34.5)	30 (21.1)	0.080
Thoracic injury, n (%)	40 (20.0)	15 (25.9)	25 (17.6)	0.210
Head trauma, n (%)	30 (15.0)	12 (20.7)	18 (12.7)	0.180
Extremity fractures, n (%)	35 (17.5)	15 (25.9)	20 (14.1)	0.080
**ISS, Median (IQR)**	21 (16–29)	25 (18–32)	18 (12–25)	<0.001
ISS ≥ 16, n (%)	80 (40.0)	30 (51.7)	50 (35.2)	0.040
Hemodynamic instability, n (%)	35 (17.5)	18 (31.0)	17 (12.0)	<0.001
Blood transfusion required, n (%)	80 (40.0)	35 (60.3)	45 (31.7)	<0.001
ICU admission, n (%)	120 (60.0)	45 (77.6)	75 (52.8)	<0.001

Baseline characteristics of the study population. Continuous variables are presented as median with interquartile range (IQR) due to non-normal distribution. Categorical variables are reported as number and percentage (%). *p*-values indicate comparisons between patients with and without major complications.

**Table 2:** Surgical Parameters and Clinical Outcomes.

Parameter	Total (n = 200)	Complications (n = 58)	No Complications (n = 142)	*p*-Value
*Surgical approach:*				
Anterior approach, n (%)	85 (42.5)	20 (34.5)	65 (45.8)	0.180
Posterior approach, n (%)	75 (37.5)	25 (43.1)	50 (35.2)	0.320
Combined approach, n (%)	40 (20.0)	13 (22.4)	27 (19.0)	0.620
Modified Stoppa, n (%)	45 (22.5)	15 (25.9)	30 (21.1)	0.520
Ilioinguinal, n (%)	34 (17.0)	8 (13.8)	26 (18.3)	0.480
Kocher-Langenbeck, n (%)	55 (27.5)	18 (31.0)	37 (26.1)	0.520
Operative time (min), mean ± SD	210 ± 60	245 ± 65	195 ± 50	<0.001
Operative time ≥ 180 min, n (%)	60 (30.0)	30 (51.7)	30 (21.1)	<0.001
Estimated blood loss (mL), median (IQR)	800 (500–1200)	1100 (700–1600)	700 (400–1000)	<0.001
Intraoperative transfusion, n (%)	80 (40.0)	35 (60.3)	45 (31.7)	<0.001
Units transfused, median (IQR)	2 (1–4)	3 (2–5)	2 (1–3)	0.020
*Implant type:*				
Reconstruction plates, n (%)	120 (60.0)	35 (60.3)	85 (59.9)	0.950
Screws only, n (%)	45 (22.5)	12 (20.7)	33 (23.2)	0.720
External fixator, n (%)	15 (7.5)	5 (8.6)	10 (7.0)	0.750
Combined fixation, n (%)	20 (10.0)	6 (10.3)	14 (9.9)	0.950
*Postoperative outcomes:*				
ICU stay (days), mean ± SD	2.3 ± 1.1	3.2 ± 1.4	1.9 ± 0.8	<0.001
ICU stay ≥ 3 days, n (%)	45 (22.5)	25 (43.1)	20 (14.1)	<0.001
Hospital LOS (days), median (IQR)	14 (10–22)	18 (12–28)	12 (9–18)	<0.001
Hospital LOS ≥ 14 days, n (%)	85 (42.5)	35 (60.3)	50 (35.2)	0.002
Readmission within 30 days, n (%)	25 (12.5)	15 (25.9)	10 (7.0)	<0.001
Reoperation within 1 year, n (%)	35 (17.5)	25 (43.1)	10 (7.0)	<0.001
30-day mortality, n (%)	8 (4.0)	5 (8.6)	3 (2.1)	0.080
1-year mortality, n (%)	12 (6.0)	8 (13.8)	4 (2.8)	0.010

Extended operative duration, increased blood loss, transfusion necessity, and prolonged intensive care unit and hospital stays demonstrated significant associations with postoperative complications (*p* < 0.05). No significant differences were found in surgical approach or implant type between groups.

**Table 3:** Postoperative Complications: Classification by Timing, Frequency, Severity, and Management.

Complication Type	n (%)	Clavien–Dindo Grade	Time to Diagnosis	Treatment Required	Classification
**Infectious complications**					
Superficial SSI	16 (8.0)	II	5–14 days	Antibiotics	**Major (≤30 days)**
Deep SSI	18 (9.0)	IIIb	7–21 days	Surgical debridement	**Major (≤30 days)**
Osteomyelitis	3 (1.5)	IVa	14–60 days	Long-term antibiotics	**Secondary (>30 days)**
Sepsis	2 (1.0)	IVa	3–10 days	ICU care	**Major (≤30 days)**
**Thromboembolic complications**					
Deep vein thrombosis	14 (7.0)	II	3–14 days	Anticoagulation	**Major (≤30 days)**
Pulmonary embolism	10 (5.0)	IVa	1–7 days	Anticoagulation/Embolectomy	**Major (≤30 days)**
Fat embolism syndrome	2 (1.0)	IVa	1–3 days	Supportive care	**Major (≤30 days)**
**Neurological complications**					
Sciatic nerve injury	2 (1.0)	IIIa	<1 day	Surgical repair	**Major (≤30 days)**
Lateral femoral cutaneous nerve injury	6 (3.0)	I–II	1–30 days	Conservative	**Major (≤30 days)**
**Orthopedic complications**					
Delayed union	4 (2.0)	IIIb	90–180 days	Bone graft	**Secondary (>30 days)**
Nonunion	7 (3.5)	IIIb	180–365 days	Revision surgery	**Secondary (>30 days)**
Malunion	8 (4.0)	IIIa	30–90 days	Osteotomy	**Secondary (>30 days)**
Avascular necrosis	5 (2.5)	IIIa	90–365 days	Surgical intervention	**Secondary (>30 days)**
Heterotopic ossification	12 (6.0)	I–IIIa	30–180 days	Surgical excision (if required)	**Secondary (>30 days)**
Implant failure	4 (2.0)	IIIb	30–180 days	Revision surgery	**Secondary (>30 days)**
Loss of reduction	6 (3.0)	IIIb	7–90 days	Revision surgery	**Major (≤30 days)**
**Other complications**					
Urological injury	3 (1.5)	IIIb	0–7 days	Surgical repair	**Major (≤30 days)**
Bowel injury	2 (1.0)	IVa	0–3 days	Surgical repair	**Major (≤30 days)**
Chronic pain syndrome	15 (7.5)	I–II	30–365 days	Pain management	**Secondary (>30 days)**
PTSD/Depression	8 (4.0)	I–II	30–365 days	Psychiatric care	**Secondary (>30 days)**

Classification was based on the timing of diagnosis: Major complications were defined as adverse events occurring within 30 days postoperatively or during hospitalization, whereas delayed union, nonunion, heterotopic ossification, chronic pain syndrome, AVN, and other late-onset findings were recorded as secondary long-term outcomes.

**Table 5:** PEARL Scoring System: Detailed Criteria and Patient Distribution.

Risk Factor	Criteria	Points	Patients (n)	Complications (%)	Definition/Notes
Age	<60 years	0	150	12	Chronological age at time of surgery
Age	≥60 years	2	50	60	Advanced age associated with comorbidities
BMI	<30 kg/m^2^	0	140	20.7	Normal/overweight patients
BMI	≥30 kg/m^2^	1	60	40	Obese patients (WHO definition)
Severe Associated Injury	Absent	0	165	21.2	Hemodynamically stable, no major organ injury
Severe Associated Injury	Present	2	35	51.4	Hemodynamic instability or major abdominal injury
Operative Duration	<180 min	0	140	21.4	Standard operative duration
Operative Duration	≥180 min	1	60	50	Prolonged surgery indicating complexity

Higher scores are associated with progressively increased complication rates, supporting the model’s clinical utility in risk stratification.

**Table 6:** Model performance metrics in derivation and validation cohorts.

Performance Metric	Derivation Cohort (n = 140)	Validation Cohort (n = 60)
**AUC (95% CI)**	0.83 (0.78–0.89)	0.78 (0.70–0.87)
**Brier Score**	0.13	0.16
**Sensitivity (%), cut-off ≥4**	84.5%	81.3%
**Specificity (%), cut-off ≥4**	75.4%	70.2%
**PPV**	54.8%	50.0%
**NPV**	93.0%	90.0%
**Accuracy (%)**	77.1%	73.3%

Discrimination and calibration performance of the PEARL score in the derivation (70% random sample) and validation (30% sample) cohorts. Discrimination was assessed using the area under the receiver operating characteristic curve (AUC), whereas calibration was evaluated using the Brier score and calibration curve). A cutoff of ≥4 points demonstrated optimal clinical balance between sensitivity and specificity.

**Table 7:** ROC Analysis and Performance Metrics for PEARL Score.

PEARL Score Cutoff	Sensitivity (%)	Specificity (%)	PPV (%)	NPV (%)	Accuracy (%)	Youden Index	LR+ (95% CI)	LR− (95% CI)
≥1	95	33.8	35.5	94.1	50	0.29	1.43 (1.25–1.64)	0.15 (0.04–0.59)
≥2	89.7	52.1	42.6	92.5	62	0.42	1.87 (1.52–2.31)	0.20 (0.09–0.44)
≥3	82.8	66.2	50	91.3	70.5	0.49	2.45 (1.84–3.26)	0.26 (0.15–0.45)
≥4	84.5	75.4	55.7	93	78	0.6	3.44 (2.41–4.91)	0.21 (0.11–0.38)
≥5	65.5	91.5	73.1	87.8	84	0.57	7.71 (4.12–14.4)	0.38 (0.26–0.55)
≥6	13.8	98.6	80	74.2	74.5	0.12	9.86 (2.31–42.1)	0.87 (0.79–0.97)

## Data Availability

The data are available upon reasonable request.

## Abbreviations

The following abbreviations are used in this manuscript:

AO/TILE	Arbeitsgemeinschaft für Osteosynthesefragen/Trauma International League of Emergency Medicine
ASA	American Society of Anesthesiologists
AUC	Area Under the Curve
BMI	Body Mass Index
CI	Confidence Interval
DVT	Deep Vein Thrombosis
ICU	Intensive Care Unit
IQR	Interquartile Range
ISS	Injury Severity Score
LOS	Length of Stay
OR	Odds Ratio
PEARL	Pelvic and Acetabular Adverse-event Risk Level
PTSD	Post-Traumatic Stress Disorder
ROC	Receiver Operating Characteristic
SSI	Surgical Site Infection
WHO	World Health Organization
